# Considering planetary health in health guidelines and health technology assessments: a scoping review protocol

**DOI:** 10.1186/s13643-024-02577-2

**Published:** 2024-06-22

**Authors:** Thomas Piggott, Maheen Raja, Charlotte T. J. Michels, Alina Herrmann, Karolina Anna Scahill, Andrea J. Darzi, Laura Jewell, KM Saif-Ur-Rahman, Hendrik Napierala, Ruben Heuer, Rebecca L. Morgan, Grigorios I. Leontiadis, Ignacio Neumann, Holger Schünemann, Fiona A. Miller

**Affiliations:** 1 Department of Health Research Methods, Evidence, and Impact, McMaster University, HSC-2C 1280 Main Street West Hamilton, Hamilton, ON L8N 3Z5 Canada; 2 Department of Family Medicine, Queens University, Kingston, Canada; 3 Knowledge Institute of the Dutch Association of Medical Specialists, Mercatorlaan 1200, Postbus 3320, Utrecht, 3502 GH The Netherlands; 4 Institute of Global Health, Heidelberg University, Heidelberg University Hospital, Im Neuenheimer Feld 324, 69120 Heidelberg, Germany; 5 Institute of General Medicine, Cologne University, Cologne University Hospital, Cologne, Germany; 6 College of Medicine and Veterinary Medicine, University of Edinburgh, 49 Little France Crescent, Edinburgh, EH16 4SB UK; 7 Evidensia Södra Djursjukhuset Kungens Kurva, Kungens Kurva, Sweden; 8 Department of Anesthesia, McMaster University, Hamilton, Canada; 9 College of Medicine, Nursing and Health Sciences, University of Galway, University Road, Galway, H91TK33 Ireland; 10 Evidence Synthesis Ireland and Cochrane Ireland, University of Galway, University Road, Galway, H91TK33 Ireland; 11 Institute of General Practice and Family Medicine, Charité-Universitätsmedizin Berlin, corporate member of Freie Universität Berlin and Humboldt Universität Zu Berlin, Charitéplatz 1, 10117 Berlin, Germany; 12 Division of Evidence-Based Medicine (dEBM), Department of Dermatology, Venereology and Allergology, Charité-Universitätsmedizin Berlin, corporate member of Freie Universität Berlin Und Humboldt Universität zu Berlin, Berlin, Germany; 13 School of Medicine, Case Western Reserve University, Cleveland, OH USA; 14 Department of Medicine, McMaster University, Hamilton, Canada; 15 School of Medicine, Universidad San Sebastián, Santiago, Chile; 16 GRADE Conosur, Universidad San Sebastián, Santiago, Chile; 17 Department of Biomedical Sciences, Humanitas University, Via Rita Levi Montalcini 4, 20090 Pieve Emanuele (Milan), Italy; 18 Institute of Health Policy, Management & Evaluation, Dalla Lana School of Public Health; Collaborative Centre for Climate, Health & Sustainable Care, University of Toronto, Toronto, ON Canada

**Keywords:** Planetary health, Health guidelines, Health technology assessments

## Abstract

**Background:**

This protocol outlines a scoping review with the objective of identifying and exploring planetary health considerations within existing health guidelines and health technology assessments (HTA). The insights gained from this review will serve as a basis for shaping future Grading of Recommendations, Assessment, Development, and Evaluations (GRADE) guidance on planetary health.

**Methods:**

We will adhere to the JBI methodology for scoping reviews. We will conduct a comprehensive search and screening of results in all languages across various databases including MEDLINE, EMBASE, CINAHL, Global Health, Health Systems Evidence, Greenfile, and Environmental Issues. Additionally, we will supplement this search with resources such as the GIN library, BIGG database, Epistemonikos, GRADE guidelines repository, GRADEpro Guideline Development Tool Database, MAGICapp, NICE website, WHO websites, and a manual exploration of unpublished relevant documents using Google incognito mode. Two independent reviewers will screen and assess the full texts of identified documents according to the eligibility criteria. The following information from each full text will be extracted: document title; first author’s name; publication year; language; document type; document as a guideline or HTA; the topic/discipline; document purpose/study objective; developing/sponsoring organization; the country in which the study/guideline/HTA report was conducted; definition of planetary health or related concept provided; types of planetary health experts engaged; study methods; suggested methods to assess planetary health; use of secondary data on planetary health outcomes; description for use of life cycle assessment; description for assessing the quality of life cycle; population/intended audience; interventions; category; applicable planetary health boundaries; consideration of social justice/global equity; phase of intervention in life cycle related to planetary health addressed; the measure of planetary health impact; impact on biodiversity/land use; one health/animal welfare mention; funding; and conflict of interest. Data analysis will involve a combination of descriptive statistics and directed content analysis, with results presented in a narrative format and displayed in tables and graphs.

**Discussion:**

The final review results will be submitted to open-access peer-reviewed journals for publication when they become available. The research findings will also be disseminated at relevant planetary health conferences and workshops.

**Systematic review registration:**

Open Science Framework (https://osf.io/3jmsa).

**Supplementary Information:**

The online version contains supplementary material available at 10.1186/s13643-024-02577-2.

## Background

Methodological developments in health guidelines and technology assessments have achieved notable progress over the past decades, supporting health decision-making, practice, and the population for which the guidance serves. However, improvements in health status and health care delivery have caused overexploitation of our planet’s resources, accompanied by pollution and the disturbance of the Earth’s vital systems [1]. These unintended consequences have driven climate change and impacted planetary health. Health system interventions make significant contributions to greenhouse gas (GHG) emissions: 5.2% of global GHGs [2], up to 10% of GHGs in the USA [3], and other negative consequences for the planet’s health [4].

There is increasing interest in planetary health as demonstrated by new journals and fields of research. This work builds on a long-standing history of considering environmental and sustainability-related considerations [5]. Planetary health extends this work to argue that human health, animal health, and the planet’s health are inextricably linked [6]. In particular, the use of Life Cycle Assessments (LCA) to assess the environmental impacts of health interventions is growing [7]. However, the term planetary health has only grown substantially within opinion pieces, rather than in published original research [8]. To date, there has been little attention paid to the integration of planetary health considerations in health technology assessments (HTAs) and health guideline decision-making. This lack of consideration and negligence of the health system, and society more broadly, towards planetary health fails to recognize the interconnectedness between human health, animal health, and the planet’s health [6]. As the climate crisis accelerates it is becoming increasingly clear that health guidelines and HTAs should consider planetary health [9].

Global guideline developers are progressively employing the Grading of Recommendations, Assessment, Development, and Evaluations (GRADE) methodology in the pursuit of guideline development. This approach, recognized for its reliability and logical underpinnings, serves as a robust framework for transitioning from evidence to the establishment of recommendations [10]. The GRADE approach yields comprehensive summaries of evidence (accompanied by evaluations of evidence certainty) and graded recommendations (entailing evaluations of recommendation strength and comprehensive evidence certainty). The GRADE approach for developing recommendations using the evidence-to-decision (EtD) framework addresses a broad range of criteria including benefits, harms, the balance of effects, certainty of evidence, resources required, cost-effectiveness, equity, acceptability, and feasibility. The EtD framework allows flexibility in the criteria considered to inform decision-making by either adding or broadening existing criteria (e.g., considering political and health system factors within acceptability and feasibility) [11], modifying criteria based on user perspective (e.g., clinical, individual, diagnostic), or by truncating the criteria [12] considered during the decision-making process. However, the current EtD criteria do not explicitly prompt consideration of planetary health.

Guidelines have recently begun incorporating environmental and planetary health considerations [13, 14]. By addressing planetary health, HTA reporters and guideline developers will be supported to assess the potential differential impacts of health interventions more comprehensively on both human health and the environment. This integration may prompt guideline developers and HTA reporters to proactively address and mitigate negative planetary health effects resulting from healthcare practices, such as the overuse of single-use plastics, carbon emissions from transportation, or other environmentally harmful impacts. This scoping review will thoroughly investigate and identify planetary health considerations in current health guidelines and HTAs. It will ultimately inform the development of future GRADE guidance on consideration of planetary health to inform decision-making [15].

## Methods

The proposed scoping review will follow the methodology outlined by the JBI (formerly Joanna Briggs Institute) for conducting scoping reviews [16, 17] and will adhere to the guidelines of the Preferred Reporting Items for Systematic Reviews and Meta-analyses extension for scoping reviews (PRISMA-ScR [18]). Recent updates of PRISMA 2020 [19], specifically the scoping review modifications as detailed in the scoping review chapter of the JBI manual on evidence synthesis [20], will also be taken into consideration (Additional file 1).

The primary purpose of this protocol is to predefine the review’s objectives, review questions, eligibility criteria, methodologies, and reporting guidelines, to ensure transparency. The protocol functions as a plan for the scoping review and is designed to mitigate potential reporting biases. Any deviations from the established protocol during the review will be clearly addressed and explained within the complete scoping review [20]. The protocol was registered in the Open Science Framework (https://osf.io/3jmsa).

A collaborative effort was initiated, bringing together a team of global experts specializing in guideline methodology and planetary health, with the purpose of contributing to this scoping review. To enhance our application of the JBI scoping review guidance and PRISMA-ScR, and to refine our screening, study selection, and data extraction, pilot testing phases were undertaken at each step. The pilot phase aimed to achieve the following objectives: (a) evaluate and enhance the methods outlined in the protocol; (b) establish and train a team of reviewers (c) establish essential group procedures; (d) create and enhance the required tools; (e) fine-tune the process and content of data extraction; and (f) ascertain the scope of our work. During the pilot, we employed an adapted approach, and after its conclusion, we further refined the methods for the proposed scoping review. This section outlines the fundamental methods employed in the proposed scoping review.

### Review question

Our goal will be to address the following review questions:

1. What are dimensions of planetary health that have been considered in health guidelines and HTAs?

2. How have guideline development methodologies suggested that health guidelines or HTAs consider the environment, climate change, or planetary health?

3. What are the methods that health guidelines and HTAs have used to incorporate evidence and assess the certainty of the evidence of planetary health outcomes?

### Eligibility criteria

#### Concept

According to The Lancet definition, planetary health is defined as “The achievement of the highest attainable standard of health, wellbeing, and equity worldwide through judicious attention to the human systems—political, economic, and social—that shape the future of humanity and the Earth's natural systems that define the safe environmental limits within which humanity can flourish. Put simply, planetary health is the health of human civilization and the state of the natural systems on which it depends” [8]. The intricate mechanisms underpinning the linkage between human health and the surrounding natural systems remain multifaceted and occasionally elusive. This discourse shifts its focal point primarily towards outcomes that transcend human-centric considerations. The concept of focus here will predominantly be on non-human-centric outcomes because past HTAs and health guidelines have historically concentrated on direct human outcomes. Thus, the imperative emerges to place substantial emphasis on the well-being of animals and the equilibrium of natural systems as measured by the planetary boundaries concept, with the recognition that the ultimate preservation of human health is fundamentally interconnected between all [21–23].

#### Context

We will include any human health guidelines or HTAs covering the breadth of clinical, health system, and public health topics, that also address planetary health outcomes (including a focus on animal health and natural systems). We will include the most recent version of the report if there are multiple versions. We will include methodological papers or handbooks that provide insight into how to address planetary health in the guideline or HTA process. We will include guidelines and HTAs published in any language, employing translation tools when studies are included beyond the languages of our team. We will not restrict eligibility based on geography or the level of government/region a guideline is focused on.

#### Types of sources

This scoping review will focus on health guidelines and HTAs that address planetary health outcomes including those that have a focus on animal health, natural systems, and the environment.

#### Exclusion criteria

In our exclusion criteria, we will exclude any studies or reports not directly related to planetary health. This includes reports or studies unrelated to health guidelines or health technology assessments. Additionally, we will exclude LCA modeling studies that do not form a part of a guideline or HTA. Furthermore, if an abstract does not explicitly reference planetary health, one health, ecosystem health, climate change, or related concepts we will exclude it from consideration for full-text screening. Concepts related to sustainability that are not relevant to environmental or planetary health, such as sustainable financing, will also be excluded. Lastly, studies focusing solely on the impacts of climate change or the environment on health (i.e., not the opposing direction of health interventions on the environment) will not be included in our review.

### Search strategy

The search strategy was developed in consultation with a health sciences librarian at McMaster University. The search will identify guidelines and HTAs that have addressed planetary health outcomes or considerations. We will complete a primary search of the literature using the following databases: Ovid MEDLINE, EMBASE, CINAHL, Global Health, Health Systems Evidence, Greenfile, and Environmental Issues. We will complement this search with the GIN international guideline library and registry of guidelines in development [24], BIGG international database of GRADE guidelines [25], Epistemonikos GRADE guidelines repository [26], GRADEpro GDT Database of GRADE EtD’s and Guidelines [27], MAGICapp [28], National Institute for Health and Care Excellence (NICE) website [29], and the World Health Organization website [30]. We will also complement this with a search for online content on Google incognito mode with similar search terms to identify any unpublished documents of relevance. In addition, the search will be further complemented by utilizing another ongoing scoping review of handbooks on guideline development, that identified 120 international guideline development handbooks by diverse organizations, to search for references to planetary health in these handbooks [31]. The search strategy for different databases has been provided in the Additional file 2: Appendix 1.

### Evidence selection

A web-based software platform Covidence (Covidence Systematic Review Software VHI, Melbourne, Australia) will be used to automatically remove duplicates and to screen the retrieved articles. The screening of citations will be carried out in two phases, both of which will go through pilot testing. Two review authors will independently screen the title and abstract of the citations based on the prespecified inclusion and exclusion criteria. A simple screening algorithm as outlined in Additional file 3: Appendix 2 will be employed. Any discrepancies will be resolved through a third reviewer.

**Fig. 1:**
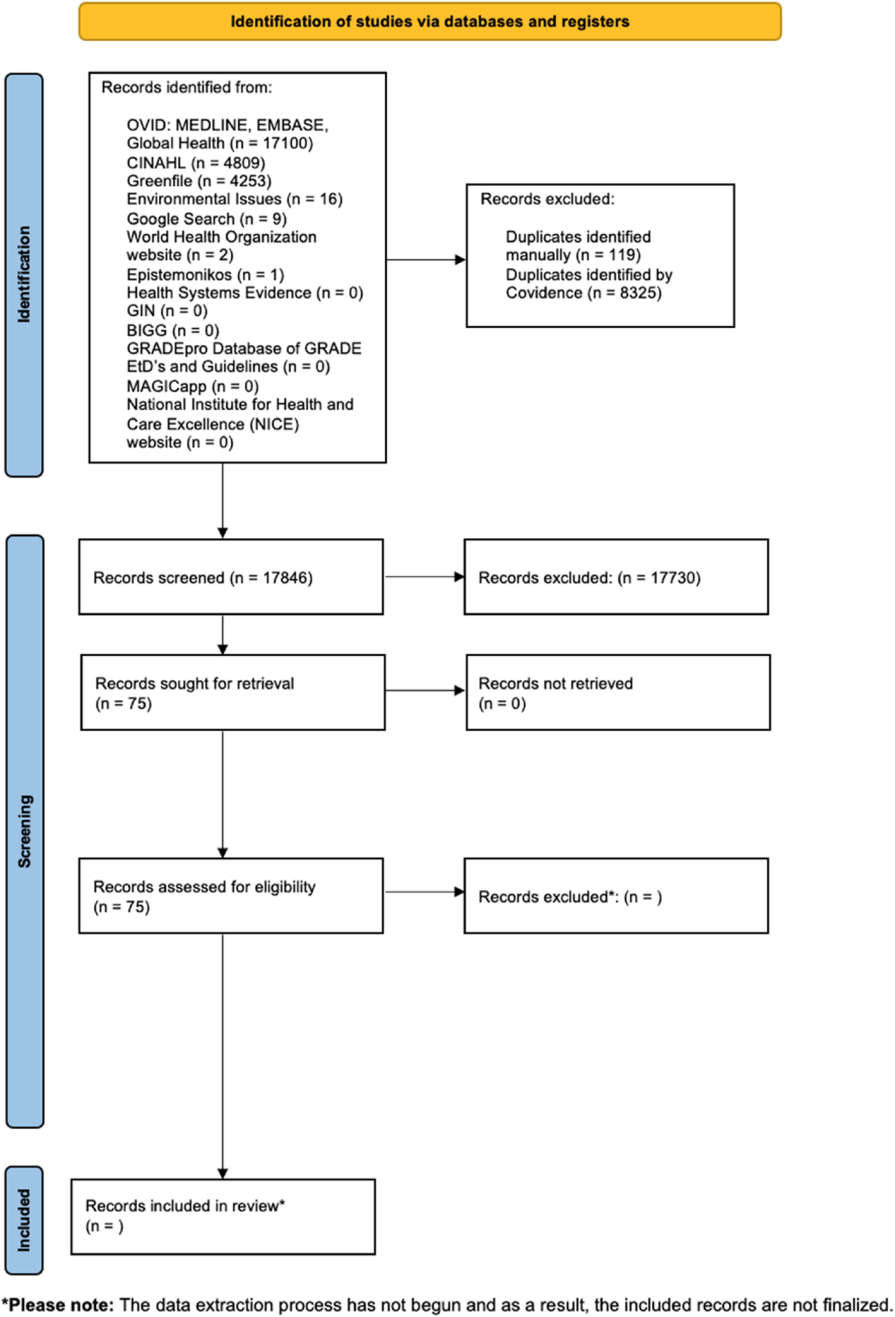
PRISMA flow diagram depicting search results before data extraction

Two reviewers will individually assess the full texts of the identified eligible documents based on the predetermined criteria for eligibility. Any conflicts will be resolved by a third reviewer. The reasons for exclusion will be documented in the full-text screening phase. A simple screening algorithm as outlined in Additional file 4: Appendix 3 will be employed. All pertinent complete guideline texts and HTA reports will be gathered and shared using an online folder. This folder will encompass associated documents and supplementary materials. The comprehensive details of the search results and the study inclusion process will be thoroughly documented in the final scoping review. This information is visually presented using a Preferred Reporting Items for Systematic Reviews and Meta-analyses (PRISMA) flow diagram [19] in Fig. 1 and will continue to be updated in the final scoping review once the data extraction process begins. Additional details can be found in the PRISMA-P checklist (see Additional file 1).

### Data extraction

Using a pilot-tested and standardized extraction form, a team of experienced reviewers will be responsible for extracting data from the guidelines and HTA reports incorporated in this scoping review. One reviewer will perform preliminary data extraction, and this will be verified by a second reviewer. Any disagreements will be resolved through discussion or involving another reviewer. The team will convene for regular meetings to address any potential concerns that might arise. These meetings will also serve the purpose of maintaining consistency and providing training to all reviewers. The primary investigator of the project will then review and refine the extracted data for each guideline and/or HTA. The following data will be extracted: document title; first author’s names; publication year; language; document type (peer review publication, guideline handbook, report, grey literature, other); whether the document is a guideline or HTA; the topic/discipline (respirology, gastroenterology, nutrition, anesthesia, other); document purpose/study objective; developing/sponsoring organization; country in which the study/guideline/HTA report was conducted (USA, UK, Canada, Australia, Germany, other); definition of planetary health or related concept provided; types of planetary health experts engaged (engineer, one health/veterinary, economist, modelling expert, geologist/earth scientist, other); study methods; suggested methods to assess planetary health (LCA, other modelling approach, use of existing databases, direct measurement, expert input on impact, other); whether the study uses secondary data on planetary health outcomes; description for use of LCA; description for assessing quality of life cycle; population/intended audience; interventions; category (human health, animal health/one health, natural systems, environment, other); applicable planetary health boundaries (climate change, change in biosphere integrity, stratospheric ozone depletion, ocean acidification, biogeochemical flows, land-system change, freshwater use, atmospheric aerosol loading, introduction of novel entities); consideration of social justice/global equity; phase of intervention in life cycle related to planetary health addressed (goods production, goods transport, patient/staff travel, diagnostic tests, facility requirements, infection prevention and control (IPAC) requirements, disposal impacts, other); measure of planetary health impact (carbon dioxide emissions, methane emissions, other greenhouse gas emission, nitrogen/phosphorous inputs, energy input, water use, waste production, monetary equivalents of impact, other); impact on biodiversity/land use and one health/animal welfare mentioned; funding (not transparent, public funding, private funding, both public and private funding, other), conflict of interest (reported or not reported).

### Data analysis and presentation

Data analysis will involve a combination of content analysis using both deductive and inductive approaches, along with the utilization of descriptive statistics. Part of the data collected, specifically the yes/no items, will be converted into quantitative form, and basic descriptive statistical methods will be employed to examine their distribution.

We will employ directed content analysis [32] to examine the textual data, with the flexibility to uncover emergent codes as well. To examine the data extracted from the guidelines/HTAs, two coders will each perform iterative rounds of analysis. Following the initial extraction, the case studies will be elucidated, and distinct themes will be identified within different categories of studies (e.g., guideline topics, and methods papers). As we go through the coding process, they will underline the important and relevant sections in the text and choose a term, phrase, or description to best capture its meaning. The codes developed during the pilot phase will serve as predetermined codes in the analysis, with additional codes being added as necessary. Codes sharing similar concepts will be organized into categories. When appropriate, explanations and examples from the text will accompany the codes or categories. The coders will not aim to quantify the frequency of code occurrences. These codes and categories will be utilized to populate the following predefined themes: goods production, goods transport, patient/staff travel, diagnostic tests, facility requirements, IPAC requirements, and disposal impacts. Additional themes will be introduced as required.

The risk of bias and the quality of the studies will be assessed for primary research papers, health guidelines/HTAs using the Risk of Bias In Non-randomized Studies of Exposures/Interventions (ROBINS I/E) tool and Appraisal of Guidelines for Research and Evaluation II (AGREE II) instrument respectively [33–35]. The quality of the theoretical or commentary papers included in the scoping review will not be assessed, as it is not typically done in scoping reviews [17, 36].

In our final conclusive assessment, we will present the results of our search using an adapted PRISMA flowchart [19]. We will also provide a concise overview of the fundamental attributes of the guidelines included, along with the outcomes of the screening procedure. Results of the analysis will be presented narratively, with codes and classifications for each topic presented in a table, as appropriate, and the quantified data presented in graphs. Any discrepancies or modifications from the established protocol will be duly documented within the final scoping review.

## Discussion and preliminary results (pilot phase)

Our team, comprised of ten reviewers, undertook a comprehensive three-part pilot exercise encompassing title and abstract screening, full-text screening, and a data extraction pilot exercise. Throughout the pilot phase, we implemented an adapted approach allowing us to address unforeseen challenges and incorporate valuable insights from our team’s collective experience. Upon the conclusion of each pilot exercise, we further refined the methods for the proposed scoping review. We provide the dates of key milestones in the scoping review process in Table 1.

**Table 1:** Review progress and timeline

Task	Date completed
Search completed	September 19th 2023
Title abstract screening pilot completed	October 26th 2023
Full-text screening pilot completed	November 16th 2023
Extraction pilot completed	January 6th 2024

### Title-abstract screening pilot exercise

In the title-abstract pilot exercise that was completed in October 2023, 50 articles were randomly selected from the compiled search results. Ten independent reviewers assessed inclusion and exclusion criteria by screening titles and abstracts of each using the Title-Abstract Screening Form (Additional file 3: Appendix 2). Reviewers’ assessments, showing variability, were compiled for discussion in a scheduled meeting to resolve discrepancies. By resolving discrepancies amongst the results, the discussions and group feedback led to refinements clarifying the inclusion/exclusion criteria, which were as follows. There would be no exclusion based on study design, but we acknowledged that a focus on guidelines or HTAs (question 2) might be limiting. Life Cycle Assessment modeling studies or other modeling studies that were not part of a guideline or HTA will be excluded. Studies lacking explicit references to planetary health, one health, ecosystem health, climate change, or related concepts in the abstract will also be excluded. Additionally, concepts unrelated to environment/planetary health, such as sustainable financing, will also be excluded, along with studies describing the impacts of climate change or the environment on health. This refinement process allowed for a more targeted and precise selection of articles for subsequent phases.

### Full-text screening pilot exercise

In the full-text pilot exercise that was completed in November 2023, 10 articles were strategically selected by the study leads to encompass both inclusion and exclusion scenarios, providing a basis for discussing common mistakes. Ten independent reviewers were tasked with evaluating the inclusion and/or exclusion criteria for all ten references by evaluating the full text using the Full-Text Screening Form (Additional file 4: Appendix 3). This form directed reviewers to assess whether the study addressed planetary health, the impact of an intervention on planetary health, and its relation to health guidelines or HTAs. Additionally, reviewers were asked to evaluate whether the study served as an example/case study of implementing planetary health considerations in guideline or HTA decision-making, and if it offered advice or suggestions, without an example/case study, on addressing planetary health considerations. The results of the exercise exhibited variability and were compiled for discussion in a scheduled meeting to reconcile discrepancies. In response to group discussions and feedback, specific criteria were clarified including what constitutes a guideline. For this study, guidelines encompass any clinical, health system, or public health guideline offering actionable statements based on evidence reviews, panel recommendations, consensus statements, position or policy statements, scientific statements, or other clear processes. Furthermore, studies focusing on policy or decision-making considerations of planetary health that lack a focus on guidelines or HTAs will be excluded.

### Data extraction pilot exercise

In the data extraction pilot exercise that was completed in January 2024, five articles meeting the inclusion criteria were selected by the study leads. Ten independent reviewers were tasked with extracting data using Additional file 5: Appendix 4: Data Extraction Form. Following data extraction, a scheduled meeting with the review team was organized to address potential inconsistencies in extraction. The form underwent significant modifications based on feedback from the team, incorporating additional items such as the topic/discipline of the study, type of planetary health experts engaged, planetary health boundaries, consideration of social justice/global equity, impact on biodiversity/land use, and impact on one health/animal welfare. These pilot exercises allowed for the refinement of the data extraction methodology for the following stages of the study (Additional file 6: Appendix 5; Additional file 7: Appendix 6).

## Conclusion

This protocol provides a description of the objectives, inclusion and exclusion criteria, methods, and analysis of a scoping review to be undertaken by an international interdisciplinary group of experts. The comprehensive findings of this scoping review will be made accessible and published in a peer-reviewed journal once they become available. To inform future guidance on planetary health within the Grading of Recommendations, Assessment, Development, and Evaluations (GRADE) criteria, the primary aim of our scoping review is to identify and examine planetary health considerations in current health guidelines and health technology assessments.

The review is currently in progress and we are targeting finalizing the analysis and submitting it in 2024. Preliminary work towards GRADE guidance on planetary health was presented at the GRADE Working Group meeting in Miami in May 2024. The scoping review actively informs the ongoing development of guidance on the consideration of planetary health in health guidelines and HTAs.

Addressing planetary health in health technology assessment (HTA) reports and guidelines will provide essential support for a more comprehensive evaluation of the potential impacts of health interventions on both human health and the environment. This holistic approach will encourage guideline developers and HTA reporters to take proactive measures in addressing and mitigating adverse effects on planetary health caused by healthcare practices, including issues such as excessive use of single-use plastics, transportation-related carbon emissions, and other environmentally detrimental impacts. Hence, promoting a healthier future for all life forms and the Earth.

### Supplementary Information

Additional file 1: PRISMA-P 2015 Checklist.

Additional file 2: Appendix 1. Search Strategy Approach.

Additional file 3: Appendix 2. Title-Abstract Screening Form.

Additional file 4: Appendix 3. Full Text Screening Guide (in Covidence).

Additional file 5: Appendix 4. Data Extraction Form.

Additional file 6: Appendix 5. Sample Included Studies for Validating Search.

Additional file 7: Appendix 6. Search Notes.

## Data Availability

The datasets used and/or analyzed during the current study are available from the corresponding author on reasonable request.

## Abbreviations

AGREE II: Appraisal of Guidelines for Research and Evaluation II
EtD: Evidence to Decision
GRADE: Grading of Recommendations, Assessment, Development, and Evaluations
GHG: Greenhouse Gas
HTA: Health Technology Assessments
IPAC: Infection Prevention and Control
LCA: Life Cycle Assessments
ROBINS I/E: Risk of Bias in Non-randomized Studies of Exposures/Interventions

## References

[1] MacNeill AJ, McGain F, Sherman JD (2021). Planetary health care: a framework for sustainable health systems. Lancet Planet Health.

[2] Romanello M, Di Napoli C, Drummond P, Green C, Kennard H, Lampard P (2022). The 2022 report of the Lancet Countdown on health and climate change: health at the mercy of fossil fuels. Lancet.

[3] Eckelman MJ, Sherman J (2016). Environmental Impacts of the U.S Health Care System and Effects on Public Health. PLoS One..

[4] Lenzen M, Malik A, Li M, Fry J, Weisz H, Pichler PP (2020). The environmental footprint of health care: a global assessment. Lancet Planet Health.

[5] Buse CG, Oestreicher JS, Ellis NR, Patrick R, Brisbois B, Jenkins AP (2018). Public health guide to field developments linking ecosystems, environments and health in the Anthropocene. J Epidemiol Community Health.

[6] Whitmee S, Haines A, Beyrer C, Boltz F, Capon AG, de Souza Dias BF (2015). Safeguarding human health in the Anthropocene epoch: report of The Rockefeller Foundation-Lancet Commission on planetary health. Lancet.

[7] Drew J, Christie SD, Rainham D, Rizan C (2022). HealthcareLCA: an open-access living database of health-care environmental impact assessments. Lancet Planet Health.

[8] Rossa-Roccor V, Acheson ES, Andrade-Rivas F, Coombe M, Ogura S, Super L (2020). Scoping review and bibliometric analysis of the term "planetary health" in the peer-reviewed literature. Front Public Health.

[9] Herrmann A, Lenzer B, Muller BS, Danquah I, Nadeau KC, Muche-Borowski C (2022). Integrating planetary health into clinical guidelines to sustainably transform health care. Lancet Planet Health.

[10] Schünemann HB, J.; Guyatt, G.; Oxman, A. GRADE Handbook https://gdt.gradepro.org/app/handbook/handbook.html2013 [

[11] Morgan RL, Kelley L, Guyatt GH, Johnson A, Lavis JN (2018). Decision-making frameworks and considerations for informing coverage decisions for healthcare interventions: a critical interpretive synthesis. J Clin Epidemiol.

[12] Alonso-Coello P, Schunemann HJ, Moberg J, Brignardello-Petersen R, Akl EA, Davoli M (2016). GRADE Evidence to Decision (EtD) frameworks: a systematic and transparent approach to making well informed healthcare choices.  1: Introduction. BMJ.

[13] Nordic Council of Ministes. Nordic Nutrition Recommendations 2023: Integrating Environmental Aspects. 2023. Available: https://norden.diva-portal.org/smash/get/diva2:1769986/FULLTEXT06.pdf.

[14] Schmiemann G, Dörks M. DEGAM S1-Guideline: Climate-conscious prescription of inhaled medications. German College of General Practitioners and Family Physicians. 2022. Available: https://register.awmf.org/assets/guidelines/053_D_Ges_fuer_Allgemeinmedizin_und_Familienmedizin/053-059eng_S1_Climate-conscious-prescription-of-inhaled-medications_2022-09.pdf.

[15] Schunemann HJ, Brennan S, Akl EA, Hultcrantz M, Alonso-Coello P, Xia J (2023). The development methods of official GRADE articles and requirements for claiming the use of GRADE - A statement by the GRADE guidance group. J Clin Epidemiol.

[16] Peters MDJ, Marnie C, Tricco AC, Pollock D, Munn Z, Alexander L (2020). Updated methodological guidance for the conduct of scoping reviews. JBI Evid Synth.

[17] Peters MDJ, Godfrey C, McInerney P, Khalil H, Larsen P, Marnie C (2022). Best practice guidance and reporting items for the development of scoping review protocols. JBI Evid Synth.

[18] Tricco AC, Lillie E, Zarin W, O'Brien KK, Colquhoun H, Levac D (2018). PRISMA Extension for Scoping Reviews (PRISMA-ScR): Checklist and Explanation. Ann Intern Med.

[19] Page MJ, McKenzie JE, Bossuyt PM, Boutron I, Hoffmann TC, Mulrow CD (2021). The PRISMA 2020 statement: An updated guideline for reporting systematic reviews. J Clin Epidemiol.

[20] Peters MDJ, Godfrey CM, McInerney P, Munn Z, Tricco AC, Khalil H. Chapter 11: Scoping Reviews (2020 version). In: Aromataris E, Munn Z (Editors). JBI Manual for Evidence Synthesis. https://synthesismanual.jbi.global: JBI; 2020.

[21] Richardson K, Steffen W, Lucht W, Bendtsen J, Cornell SE, Donges JF (2023). Earth beyond six of nine planetary boundaries. Sci Adv..

[22] Steffen W, Richardson K, Rockstrom J, Cornell SE, Fetzer I, Bennett EM (2015). Sustainability Planetary boundaries: guiding human development on a changing planet. Science..

[23] Rockström  J, Steffen W, Noone K, Persson Å, Chapin, III FS, Lambin E (2009). Planetary Boundaries: Exploring the Safe Operating Space for Humanity. Ecol Soc.

[24] GIN International Guideline Library and Registry of Guidelines in Development [Available from: https://guidelines.ebmportal.com/.

[25] BIGG International Database of GRADE guidelines [Available from: https://sites.bvsalud.org/bigg/en/biblio/. 10.1016/j.lana.2021.100099 PMC8863112 35233554

[26] Epistemonikos GRADE Guidelines Repository [Available from: https://www.epistemonikos.org/en/groups/grade_guideline.

[27] GRADEpro GDT Database of GRADE EtD’s and Guidelines [Available from: https://guidelines.gradepro.org/.

[28] MAGICapp [Available from: https://app.magicapp.org/#/guidelines.

[29] National Institute for Health and Care Excellence (NICE) [Available from: https://www.nice.org.uk/guidance/published?type=ph.

[30] World Health Organization [Available from: https://www.who.int/publications/i.

[31] Khabsa J, Nour Eldine M, Yaacoub S, El-Khoury R, El Yaman N, Wiercioch W, Schünemann HJ, Akl EA. (submitted). Guideline development methods based on a survey of handbooks: 1. Introduction to the series.

[32] Hsieh HF, Shannon SE (2005). Three approaches to qualitative content analysis. Qual Health Res.

[33] Sterne JA, Hernan MA, Reeves BC, Savovic J, Berkman ND, Viswanathan M (2016). ROBINS-I: a tool for assessing risk of bias in non-randomised studies of interventions. BMJ.

[34] ROBINS-E Development Group (Higgins J MR, Rooney A, Taylor K, Thayer K, Silva R, Lemeris C, Akl A, Arroyave W, Bateson T, Berkman N, Demers P, Forastiere F, Glenn B, Hróbjartsson A, Kirrane E, LaKind J, Luben T, Lunn R, McAleenan A, McGuinness L, Meerpohl J, Mehta S, Nachman R, Obbagy J, O'Connor A, Radke E, Savović J, Schubauer-Berigan M, Schwingl P, Schunemann H, Shea B, Steenland K, Stewart T, Straif K, Tilling K, Verbeek V, Vermeulen R, Viswanathan M, Zahm S, Sterne J). Risk Of Bias In Non-randomized Studies - of Exposure (ROBINS-E). 2023.

[35] Brouwers MC, Kho ME, Browman GP, Burgers JS, Cluzeau F, Feder G (2010). AGREE II: advancing guideline development, reporting, and evaluation in health care. Prev Med.

[36] Pollock D, Davies EL, Peters MDJ, Tricco AC, Alexander L, McInerney P (2021). Undertaking a scoping review: A practical guide for nursing and midwifery students, clinicians, researchers, and academics. J Adv Nurs.

