# Supplementary material for: Considering planetary health in health guidelines and health technology assessments: a scoping review protocol
Source: Syst Rev. 2024 Jun 22;13:163. doi: 10.1186/s13643-024-02577-2 (PMC11193899; doi:10.1186/s13643-024-02577-2)
Supplement: Supplementary file 2 — Additional file 2: Appendix 1. Search Strategy Approach. [file 13643_2024_2577_MOESM2_ESM.docx]

**Appendix 1: Search Strategy Approach**

Considering Planetary Health in Health Guidelines: A Scoping Review

OVID: MEDLINE, EMBASE, Global Health

| Search Number | Query | Results |
| --- | --- | --- |
| #1 | exp planetary health/ | 474 |
| #2 | (planet* health or ecosystem* health or one health or climate change* health or environment* health or sustainab* or carbon footprint).ab,ti. | 388748 |
| #3 | 1 or 2 | 388877 |
| #4 | exp practice guideline/ | 746539 |
| #5 | (((((((guideline* or consensus* or health plan* guideline or position statement? or policy statement? or scientific statement? or recommendation? or clinical practice guideline or clinical guideline* or health) adj2 guideline*) or practice) adj2 guideline*) or clinical) adj2 guideline*) or practice pattern*).ab,ti. | 1334958 |
| #6 | 4 or 5 | 1734082 |
| #7 | exp biomedical technology assessment/ | 29427 |
| #8 | (health tech* assessment or health* technology assessment or healthy technology eval* or health* technology analysis or biomedical technology assess* or health econ* evaluation or HTA).ab,ti. | 24957 |
| #9 | 7 or 8 | 46809 |
| #10 | 6 or 9 | 1775488 |
| #11 | 3 and 10 | 17100 |

CINAHIL Search Results

| Search Number | Query | Results |
| --- | --- | --- |
| S1 | (MH "Ecosystem+") | (10,814) |
| S2 | TI (planet* health or ecosystem* health or one health or climate change* health or environment* health or sustainab* or carbon footprint) OR AB (planet* health or ecosystem* health or one health or climate change* health or environment* health or sustainab* or carbon footprint) | (37,837) |
| S3 | S1 or S2 | (48,366) |
| S4 | (MH "Practice Guidelines") | (85,739) |
| S5 | TI (guideline* or consensus* or health plan* guideline or position statement? or policy statement? or scientific statement? or recommendation? or clinical practice guideline or clinical guideline* or health N2 guideline* or practice N2 guideline* or clinical N2 guideline* or practice pattern*) OR AB (guideline* or consensus* or health plan* guideline or position statement? or policy statement? or scientific statement? or recommendation? or clinical practice guideline or clinical guideline* or health N2 guideline* or practice N2 guideline* or clinical N2 guideline* or practice pattern*) | (349,636) |
| S6 | S4 OR S5 | (389,684) |
| S7 | (MH "Health Impact Assessment") | (867) |
| S8 | TI (health tech* assessment or health* technology assessment or healthy technology eval* or health* technology analysis or biomedical technology assess* or health econ* evaluation or HTA) OR AB (health tech* assessment or health* technology assessment or healthy technology eval* or health* technology analysis or biomedical technology assess* or health econ* evaluation or HTA) | (4,458) |
| S9 | S7 OR S8 | (5,316) |
| S10 | S6 OR S9 | (393,949) |
| S11 | S3 AND S10 | (4,810) |

Greenfile

|  | Search Term | Results |
| --- | --- | --- |
|  | (TI ((planet* health OR ecosystem* health OR one health OR climate change* health OR environment* health OR sustainab* OR carbon footprint))) OR (AB ((planet* health OR ecosystem* health OR one health OR climate change* health OR environment* health OR sustainab* OR carbon footprint))) | 86,043 |
|  | (TI ((guideline* OR consensus* OR health plan* guideline OR position statement? OR policy statement? OR scientific statement? OR recommendation? OR clinical practice guideline OR clinical guideline* OR health N2 guideline* OR practice N2 guideline* OR clinical N2 guideline* OR practice pattern*))) OR (AB ((guideline* OR consensus* OR health plan* guideline OR position statement? OR policy statement? OR scientific statement? OR recommendation? OR clinical practice guideline OR clinical guideline* OR health N2 guideline* OR practice N2 guideline* OR clinical N2 guideline* OR practice pattern*))) | 29,006 |
|  | (TI ((health tech* assessment OR health* technology assessment OR healthy technology eval* OR health* technology analysis OR biomedical technology assess* OR health econ* evaluation OR HTA))) OR (AB ((health tech* assessment OR health* technology assessment OR healthy technology eval* OR health* technology analysis OR biomedical technology assess* OR health econ* evaluation OR HTA))) | 25 |
|  | ((TI ((health tech* assessment OR health* technology assessment OR healthy technology eval* OR health* technology analysis OR biomedical technology assess* OR health econ* evaluation OR HTA))) OR (AB ((health tech* assessment OR health* technology assessment OR healthy technology eval* OR health* technology analysis OR biomedical technology assess* OR health econ* evaluation OR HTA)))) OR ((TI ((guideline* OR consensus* OR health plan* guideline OR position statement? OR policy statement? OR scientific statement? OR recommendation? OR clinical practice guideline OR clinical guideline* OR health N2 guideline* OR practice N2 guideline* OR clinical N2 guideline* OR practice pattern*))) OR (AB ((guideline* OR consensus* OR health plan* guideline OR position statement? OR policy statement? OR scientific statement? OR recommendation? OR clinical practice guideline OR clinical guideline* OR health N2 guideline* OR practice N2 guideline* OR clinical N2 guideline* OR practice pattern*)))) | 29,031 |
|  | (((TI ((health tech* assessment OR health* technology assessment OR healthy technology eval* OR health* technology analysis OR biomedical technology assess* OR health econ* evaluation OR HTA))) OR (AB ((health tech* assessment OR health* technology assessment OR healthy technology eval* OR health* technology analysis OR biomedical technology assess* OR health econ* evaluation OR HTA)))) OR ((TI ((guideline* OR consensus* OR health plan* guideline OR position statement? OR policy statement? OR scientific statement? OR recommendation? OR clinical practice guideline OR clinical guideline* OR health N2 guideline* OR practice N2 guideline* OR clinical N2 guideline* OR practice pattern*))) OR (AB ((guideline* OR consensus* OR health plan* guideline OR position statement? OR policy statement? OR scientific statement? OR recommendation? OR clinical practice guideline OR clinical guideline* OR health N2 guideline* OR practice N2 guideline* OR clinical N2 guideline* OR practice pattern*))))) AND ((TI ((planet* health OR ecosystem* health OR one health OR climate change* health OR environment* health OR sustainab* OR carbon footprint))) OR (AB ((planet* health OR ecosystem* health OR one health OR climate change* health OR environment* health OR sustainab* OR carbon footprint)))) | 4,253 |

Epistemonikos:

Title/abstract: (title:((((((((guideline* OR consensus* OR health plan* guideline OR position statement? OR policy statement? OR scientific statement? OR recommendation? OR clinical practice guideline OR clinical guideline* OR health) adj2 guideline*) OR practice) adj2 guideline*) OR clinical) adj2 guideline*) OR practice pattern*)) OR abstract:((((((((guideline* OR consensus* OR health plan* guideline OR position statement? OR policy statement? OR scientific statement? OR recommendation? OR clinical practice guideline OR clinical guideline* OR health) adj2 guideline*) OR practice) adj2 guideline*) OR clinical) adj2 guideline*) OR practice pattern*))) OR (title:((health tech* assessment OR health* technology assessment OR healthy technology eval* OR health* technology analysis OR biomedical technology assess* OR health econ* evaluation OR HTA)) OR abstract:((health tech* assessment OR health* technology assessment OR healthy technology eval* OR health* technology analysis OR biomedical technology assess* OR health econ* evaluation OR HTA)))

AND

Title/Abstract: (planet* health or ecosystem* health or one health or climate change* health or environment* health or sustainab* or carbon footprint)
