# Supplementary material for: Considering planetary health in health guidelines and health technology assessments: a scoping review protocol
Source: Syst Rev. 2024 Jun 22;13:163. doi: 10.1186/s13643-024-02577-2 (PMC11193899; doi:10.1186/s13643-024-02577-2)
Supplement: Supplementary file 3 — Additional file 3: Appendix 2. Title-Abstract Screening Form. [file 13643_2024_2577_MOESM3_ESM.docx]

**Appendix 2: Title-Abstract Screening Form**

Considering Planetary Health in Health Guidelines: A Scoping Review

Title and Abstract Screening Guide

1. Is this study about planetary health [see definition in protocol]?

- No => Exclude
- Yes or Uncertain => Next question

1. Is this study related to health guidelines or health technology assessments (HTAs)?

- No => Exclude
- Yes or Uncertain => Next question

1. Does the abstract reference provide advice or suggestions on how to address planetary health considerations by a guideline?

- No or Uncertain => Next question
- Yes => Include

1. Does the abstract reference advice or suggestions on how to address planetary health considerations by a HTAs?

- No => Exclude
- Yes or Uncertain => Include

**Notes:**

- We will not exclude by language or impose any language restrictions;
- We will not exclude by study design, however, focusing on guidelines or HTAs (question 2) may limit; e.g. we will exclude literature/systematic reviews that do not provide recommendations;
- We will exclude Life Cycle Assessment modelling studies or other modelling studies that are not part of a guideline or HTA (study that evaluates nutritional guidance with regards to environmental impacts but is not focused on methodology for how to do this, or integrates this directly into the guideline or HTA);
- We will exclude studies that do not have an explicit reference to planetary health, one health, ecosystem health or climate change in the abstract;
- If you come across duplicate studies indicate that it was excluded as it was duplicate even if the study is excluded (to keep track of the duplicate numbers);
- We will exclude concepts related to sustainability that is not environment/planetary health related (e.g. sustainable financing);
- We will exclude studies that describe impacts of climate change or the environment on health;
- We will exclude studies that are focused on policy or decision-making consideration of planetary health that do not include a focus on guidelines or HTAs;
