# Supplementary material for: Considering planetary health in health guidelines and health technology assessments: a scoping review protocol
Source: Syst Rev. 2024 Jun 22;13:163. doi: 10.1186/s13643-024-02577-2 (PMC11193899; doi:10.1186/s13643-024-02577-2)
Supplement: Supplementary file 4 — Additional file 4: Appendix 3. Full Text Screening Guide (in Covidence). [file 13643_2024_2577_MOESM4_ESM.docx]

**Appendix 3:** **Full Text Screening Guide (in Covidence)**

Considering Planetary Health in Health Guidelines: A Scoping Review

Study ID:

First author:

Year:

Screener initials:

1. Is this study about planetary health [see definition in protocol]?
    *According to The Lancet definition, planetary health it is defined as, “The achievement of the highest attainable standard of health, wellbeing, and equity worldwide through judicious attention to the human systems—political, economic, and social—that shape the future of humanity and the Earth's natural systems that define the safe environmental limits within which humanity can flourish. Put simply, planetary health is the health of human civilisation and the state of the natural systems on which it depends”*

- No => Exclude
- Yes or Uncertain => Next question

1. Is this study about the impact of an intervention on planetary health?

- No => Exclude
- Yes or Uncertain => Next question

1. Is this study related to health guidelines or health technology assessments (HTAs)?

- No => Exclude
- Yes or Uncertain => Next question

1. Is this study an example/case study of implementing planetary health considerations as part of guideline or HTA decision-making?

- No or Uncertain => Next question
- Yes => Include

1. Does the study offer advice or suggestions, without an example/case study, on how to address planetary health considerations by a guideline or HTA?

- No => Exclude (however, if the is a systematic review, scoping review, or literature review that does not offer advice or suggestions we will screen references for inclusion)
- Yes => Include

**Reasons for exclusion:**

- 1: Study not about planetary health
- 2: Study not about impact of an intervention on planetary health (Wrong direction of impact: Report related to the impact of planetary health/environmental health/climate change on human health, not the impact of an intervention on planetary health)
- 3: Study not related to health guidelines or health technology assessments
- 4: Study does provide advice or suggestions on consideration of planetary health in guidelines or HTA
- 5: Full-text unretrievable [leads will assess]
- 6: Duplicate study [leads will assess]

**Notes:**

- We will not exclude by language or impose any language restrictions;
- We will not exclude by study design, however, focusing on guidelines or HTAs (question 2) may limit; e.g. we will exclude literature/systematic reviews that do not provide recommendations;
- Guidelines may include any clinical, health system, or public health guideline that makes actionable statements based on evidence reviews and panel recommendations, consensus statements, position or policy statements, scientific statements or other clear process.
- We will exclude Life Cycle Assessment modelling studies or other modelling studies that are not part of a guideline or HTA (study that evaluates nutritional guidance with regards to environmental impacts but is not focused on methodology for how to do this, or integrates this directly into the guideline or HTA);
- We will exclude studies that do not have an explicit reference to planetary health, one health, ecosystem health or climate change in the abstract;
- We will exclude duplicate studies. If you come across duplicate studies indicate that it was excluded as it was duplicate even if the study is excluded (to keep track of the duplicate numbers);
- We will exclude concepts related to sustainability that is not environment/planetary health related (e.g. sustainable financing);
- We will exclude studies that describe impacts of climate change or the environment on health;
- We will exclude studies that are focused on policy or decision-making consideration of planetary health that do not include a focus on guidelines or HTAs;
