# Supplementary material for: Considering planetary health in health guidelines and health technology assessments: a scoping review protocol
Source: Syst Rev. 2024 Jun 22;13:163. doi: 10.1186/s13643-024-02577-2 (PMC11193899; doi:10.1186/s13643-024-02577-2)
Supplement: Supplementary file 5 — Additional file 5: Appendix 4. Data Extraction Form. [file 13643_2024_2577_MOESM5_ESM.docx]

**Appendix 4: Data Extraction Form**

Considering Planetary Health in Health Guidelines: A Scoping Review

| *Item* | *Extracted information* | | | | | | | | | | | | | | | | | | | | | | | | | | | | | | | | | | | |
| --- | --- | --- | --- | --- | --- | --- | --- | --- | --- | --- | --- | --- | --- | --- | --- | --- | --- | --- | --- | --- | --- | --- | --- | --- | --- | --- | --- | --- | --- | --- | --- | --- | --- | --- | --- | --- |
| Title |  | | | | | | | | | | | | | | | | | | | | | | | | | | | | | | | | | | | |
| First author’s last name |  | | | | | | | | | | | | | | | | | | | | | | | | | | | | | | | | | | | |
| Publication year |  | | | | | | | | | | | | | | | | | | | | | | | | | | | | | | | | | | | |
| Language |  | | | | | | | | | | | | | | | | | | | | | | | | | | | | | | | | | | | |
| Document type | Peer review publication | | | | | | | Guideline handbook | | | | | | | | Report | | | | | | Grey literature | | | | | | Other | | | | | | | | |
| Is this a guideline? | Yes | | | | | | | | | | | | | | | No | | | | | | | | | | | | | | | | | | | | |
| Is this a health technology assessment (HTA)? | Yes | | | | | | | | | | | | | | | No | | | | | | | | | | | | | | | | | | | | |
| Topic/ discipline | Respirology | | | | | | | Gastroenterology | | | | | | | | | | Nutrition | | | | | | | | Anesthesia | | | | | | | Other | | | |
| Document purpose/ Study objective |  | | | | | | | | | | | | | | | | | | | | | | | | | | | | | | | | | | | |
| Developing/ Sponsoring organization |  | | | | | | | | | | | | | | | | | | | | | | | | | | | | | | | | | | | |
| Country in which the study was conducted | United States | | | | | UK | | | | | | Canada | | | | | | | Australia | | | | | | | | Germany | | | | | Other | | | | |
| Definition of planetary health or related concept provided |  | | | | | | | | | | | | | | | | | | | | | | | | | | | | | | | | | | | |
| Types of planetary health experts engaged | Engineer | | | | | One health/ veterinary | | | | | | Economist | | | | | | | Modelling expert | | | | | | | | Geology/ Earth science | | | | | Other | | | | |
| Study methods |  | | | | | | | | | | | | | | | | | | | | | | | | | | | | | | | | | | | |
| Suggested methods to assess planetary health | Life cycle assessment | | | | | Other modelling approach | | | | | | Use of existing databases (e.g. estimates on carbon emission) | | | | | | | Direct measurement | | | | | | | | Expert input on impact | | | | | Other | | | | |
| Does this study use secondary data on planetary health outcomes? | Yes | | | | | | | | | | | | | | | | | | No | | | | | | | | | | | | | | | | | |
| Description for use of life cycle assessment (if applicable) |  | | | | | | | | | | | | | | | | | | | | | | | | | | | | | | | | | | | |
| Description for assessing quality of life cycle assessment (if applicable) |  | | | | | | | | | | | | | | | | | | | | | | | | | | | | | | | | | | | |
| Population(s) / Intended audience |  | | | | | | | | | | | | | | | | | | | | | | | | | | | | | | | | | | | |
| Intervention(s) |  | | | | | | | | | | | | | | | | | | | | | | | | | | | | | | | | | | | |
| Outcome - category | Human health | | | | | | Animal health | | | | | | | | Natural systems | | | | | | | | Environment | | | | | | | | Other | | | | | |
| Outcome – applicable planetary health boundaries | Climate change | Change in biosphere integrity (biodiversity loss and species extinction) | | | | | | | Stratospheric ozone depletion | | | Ocean acidification | | | | | Biogeochemical flows (phosphorous and nitrogen cycle) | | | | Land-system change (e.g. deforestation) | | | | | | Freshwater use | | | Atmospheric aerosol loading (microscopic particles in the atmosphere that affect climate change and living organisms) | | | | | Introduction of novel entities | |
| Outcome - consideration of social justice/global equity | Yes | | | | | | | | | | | | | | | | | | No | | | | | | | | | | | | | | | | | |
| Planetary health/related concept included to phase of intervention in life cycle | Goods production | | | Goods transport | | | | | | Patient/ staff travel | | | | Diagnostic tests | | | | | Facility requirements | | | | | | IPAC requirements | | | | Disposal impacts | | | | | Other | | |
| Outcome – measure of planetary health impact | CO2 emissions (tons of carbon) | Methane emissions | | | | | | | Other greenhouse gas emission | | | Nitrogen/phosphorous inputs | | | | | Energy input (kilowatt, megawatt) | | | | Water use | | | | | | Waste production (weight, e.g. kilograms) | | | Monetary equivalents of impact | | | | | Other | |
| Impact on biodiversity/land use mentioned? | Yes | | | | | | | | | | | | | | | | | | No | | | | | | | | | | | | | | | | | |
| Impact on one health/animal welfare mentioned? | Yes | | | | | | | | | | | | | | | | | | No | | | | | | | | | | | | | | | | | |
| Was risk of bias assessed for the included evidence/studies? | Yes | | | | | | | | | | | | | | | | | | No | | | | | | | | | | | | | | | | | |
| How was the planetary health evidence included used for the guideline or HTA decision-making if applicable? |  | | | | | | | | | | | | | | | | | | | | | | | | | | | | | | | | | | | |
| Funding | Not transparent | | | | | | Public funding | | | | | | | | Private funding | | | | | | | | Both public and private | | | | | | | | Other | | | | | |
| Conflict of interest | Reported | | | | | | | | | | | | | | | Not reported | | | | | | | | | | | | | | | | | | | | |
|  |  | |  | |  | | |  | | |  | |  | | |  | |  | |  | |  | |  | |  | |  | | | | |  | | |  |
