# Supplementary material for: Considering planetary health in health guidelines and health technology assessments: a scoping review protocol
Source: Syst Rev. 2024 Jun 22;13:163. doi: 10.1186/s13643-024-02577-2 (PMC11193899; doi:10.1186/s13643-024-02577-2)
Supplement: Supplementary file 6 — Additional file 6: Appendix 5. Sample Included Studies for Validating Search. [file 13643_2024_2577_MOESM6_ESM.docx]

**Appendix 5: Sample Included Studies for Validating Search**

Considering Planetary Health in Health Guidelines: A Scoping Review

1. Herrmann A, Lenzer B, Muller BS, Danquah I, Nadeau KC, Muche-Borowski C, et al. Integrating planetary health into clinical guidelines to sustainably transform health care. Lancet Planet Health. 2022;6(3):e184-e5.

2. Heuer R, Nast A. [Sustainable prescription and implementation practices in clinical practice guidelines]. Dermatologie (Heidelb). 2023;74(1):34-40.

3. MacNeill AJ, McGain F, Sherman JD. Planetary health care: a framework for sustainable health systems. Lancet Planet Health. 2021;5(2):e66-e8.

**German Guidelines:**

1. Breitbart E, Bauer A, Diepgen T. S3 guideline prevention of skin cancer 2021 [Available from: <https://register.awmf.org/de/leitlinien/detail/032-052OL>.

2. Jendyk R, Maisel P. S1 guideline on heat-related health disorders in general practice German Society for General Medicine and Family Medicine2020 [Available from: <https://register.awmf.org/de/leitlinien/detail/053-052>.

3. Schmiemann G, Dorks M. S1 guideline on climate-conscious prescription of inhalants German Society for General Medicine and Family Medicine2022 [Available from: <https://register.awmf.org/de/leitlinien/detail/053-059>.

4. Measures for the prevention and control of SARS-CoV-2 transmission in schools

S3-Leitlinie Maßnahmen zur Prävention und Kontrolle der SARS-CoV-2-Übertragung in Schulen - Lebende Leitlinie (Living Guideline) <https://register.awmf.org/de/leitlinien/detail/027-076>
