# Supplementary material for: Considering planetary health in health guidelines and health technology assessments: a scoping review protocol
Source: Syst Rev. 2024 Jun 22;13:163. doi: 10.1186/s13643-024-02577-2 (PMC11193899; doi:10.1186/s13643-024-02577-2)
Supplement: Supplementary file 7 — Additional file 7: Appendix 6. Search Notes. [file 13643_2024_2577_MOESM7_ESM.docx]

**Appendix 6: Search Notes**

Considering Planetary Health in Health Guidelines: A Scoping Review

| Database | Date | Who Completed |  |
| --- | --- | --- | --- |
| OVID: MEDLINE, EMBASE, Global Health | Sept 13 2023 | Maheen Raja | 17,100 |
| CINAHL | Sept 13 2023 | Maheen Raja | 4,809 |
| Health Systems Evidence | Sept 13 | Maheen Raja | 0 |
| GIN | Sept 13 | Maheen Raja | 0 |
| BIGG | Sept 13 | Maheen Raja | 0 |
| Epistemonikos | Sept 13 | Maheen Raja | 1 |
| GRADEpro Database of GRADE EtD’s and Guidelines | Sept 13 | Maheen Raja | 0 |
| MAGICapp | Sept 13 | Maheen Raja | 0 |
| National Institute for Health and Care Excellence (NICE) website | Sept 13 | Maheen Raja | 0 |
| World Health Organization website | Sept 13 | Maheen Raja | 2 |
| Google Search (incognito) | Sept 13 | Maheen Raja | 9 |
| Greenfile | Sept 19 * restrict date to Sept 13 | Maheen Raja | 4,253 |
| Environmental Issues | Sept 19 * restrict date to Sept 13 | Maheen Raja | 16 |
